# Effects of BTEX on the Removal of Acetone in a Coaxial Non-Thermal Plasma Reactor: Role Analysis of the Methyl Group

**DOI:** 10.3390/molecules23040890

**Published:** 2018-04-05

**Authors:** Liyuan Hou, Xiang Li, Deyuan Xie, Haining Wang

**Affiliations:** School of Space and Environment, Beihang University, Beijing 100191, China; houliyuan@126.com (L.H.); xiedeyuan@126.com (D.X.); wanghaining@buaa.com (H.W.)

**Keywords:** non-thermal plasma, byproducts, methyl group, acetone, BTEX

## Abstract

The removal of acetone and benzene series (BTEX) under individual and concurrent conditions is carried out in a coaxial nonthermal plasma (NTP) reactor. The results show that the benzene series has a significant negative impact on acetone conversion and CO_2_ selectivity under NTP treatment. Furthermore, it is found that *p*-xylene significantly promotes CO_x_ selectivity under co-treatment with acetone because of greater CO generation. Based on the results of transient FTIR, MS, and GC-MS, it is seen that quantities of formic acid, formaldehyde, and ring-opening byproducts from benzene series decomposition are reduced, while quantities of aromatic byproducts with carboxyl, phenolic, and aldehyde groups on the benzene ring increase under coexistence conditions. With the help of theoretical calculation and kinetic analysis, hydrogen abstraction from the methyl group and active hydroxyl radical consumption are proposed as critical factors in the BTEX inhibition effect on acetone degradation.

## 1. Introduction

As an important predecessor of submicron particles (PM2.5) and ozone pollution in the atmosphere, volatile organic compounds (VOCs) emitted from industrial manufacturing have received increasing attention in recent years [1,2]. Among the VOCs, the benzene series (BTEX) and acetone have always been simultaneously utilized in paints, solvents, and raw materials in the chemical and printing industry [3,4,5]. Exposure usually causes a number of environment-related health problems, including dizziness, nausea, organ damage, and even cancer [6]. Thus, developing suitable abatement methods for BTEX/acetone emission control is urgent and significant. Conventional technologies for VOC removal include adsorption, thermal combustion, chemical absorption, and catalytic oxidation. However, such methods are not sufficiently cost-effective or suitable for removal of dilute concentrations (<1000 ppm) of contaminants under high space velocity because of low efficiencies and high energy consumption [7]

In recent years, non-thermal plasma (NTP) has been regarded as an energy-saving, efficient, and promising method for low-concentration VOC abatement due to its environmentally-friendly nature, fast ignition response, and strong oxidative degradation ability [8,9]. At room temperature, quantities of highly energetic electrons and reactive species generated in the discharge area trigger a cascade of plasma chemistry reactions, resulting in the removal of pollutants [10,11,12]. Several studies on BTEX removal, side product analysis, and degradation mechanisms by NTP have been reported over the past few years. Satoh et al. explored the effect of O_2_ proportion in carrier gas on the removal of benzene by different manners of discharge. The results shows that at low oxygen concentration, the byproducts are primarily C_2_H_2_, HCN, NO, and HCOOH, while only HCOOH is found at high oxygen concentrations [13]. Stefan et al. investigated the degradation of cyclohexene and BTEX in an NTP air purifying system, and the degradation efficiency order of benzene (<1%) < xylene (3%) ≈ ethylbenzene < toluene (11%) ≈ cyclohexene was found [14]. Our previous research indicates that the conversion of low-concentration benzene, toluene, and *p*-xylene increases from 2%, 19%, and 49%, respectively, at an energy density (ED) of 10 J·L^−1^ under positive corona discharge for BTEX [15]. Unlike BTEX, there are few reports focused on acetone degradation by NTP. In research, monolithic ceramic catalysts and CuO/γ-Al_2_O_3_ are often added into the plasma reactor or after the reaction to intensify the decomposition of acetone plasma [16,17]. With respect to byproduct investigation, Narengerile et al. evaluated the acetone decomposition efficiency in DC water plasmas at atmospheric pressure. It was found that aqueous acetone can be successfully decomposed into H_2_, CO_2_, CO, and CH_4_, but unwanted byproducts, such as HCOOH and HCHO, also form [18].

However, some limitations on the study of acetone and BTEX removal by NTP still exist as follows. Firstly, acetone, as a representative of oxygen-containing VOCs, is hardly decomposed, but has the highest emission limit (100 mg·m^−3^) among VOCs from petrochemical industry emissions [19]. However, few studies have focused on its removal effectiveness, especially at low concentrations. Secondly, in general typical industrial emissions contain a blend of VOCs [20,21]. However, research often focuses on single-component VOCs rather than mixtures of VOCs, which is not in accordance with real emission conditions. Thirdly, though BTEX concentrations are far below those of acetone in real life, their compounds are characteristic constituents of the gaseous effluents of wastewater treatments in petrochemical plants [19]. Whether there is an interaction between acetone and BTEX when treated together remains unknown.

Accordingly, this study focuses on a mixture of acetone and BTEX degradation using corona discharge that aims at investigating possible influencing mechanisms in removal efficiency and COx selectivity under NTP treatment. In addition, the impact of BTEX types on NO_x_, O_3_, organic byproduct formation, and the acetone degradation pathway are also studied by experimental and theory calculations in this paper.

## 2. Experimental

### 2.1. Experimental System

**Figure 1:**
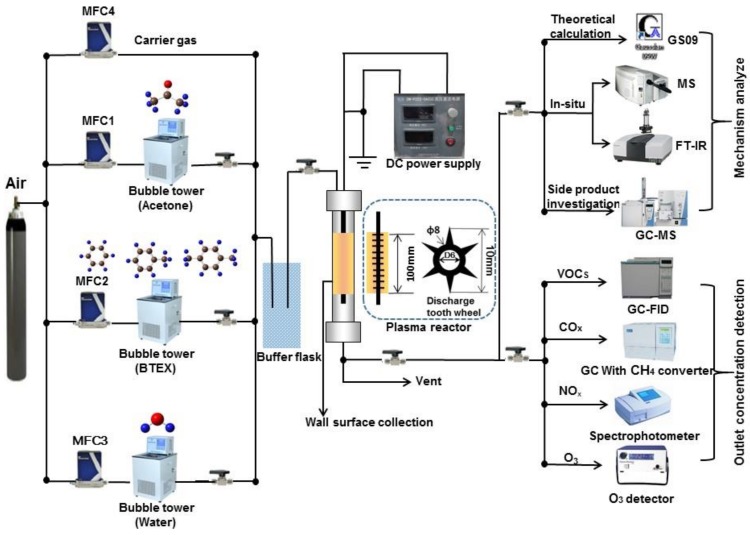
Schematic diagram of the experimental system.

The experimental system is shown in Figure 1. It consists of a coaxial link tooth wheel-cylinder plasma reactor with a 25 kV/5 mA negative direct current (DC) high voltage power supply, reaction gas supply, and analytical instrumentation. It is a stainless steel cylinder with an inner diameter of 42 mm and a length of 300 mm that serves as the ground electrode of the plasma reactor. The high voltage electrode is a stainless steel rod (o.d. 6 mm) through which 10 discharge teeth wheels are linked with a space interval of 10 mm, while each wheel has six discharge cusps. The effective discharge length and discharge intervals are 100 and 16 mm, respectively. The visual appearance of the discharge is that of a gleamy plasma column completely filling the inter-electrode space, representing a streamer-like corona discharge.

### 2.2. Experimental Methods

Experiments with both the single acetone and acetone with BTEX (benzene, toluene, or *p*-xylene) were conducted in this study. Gaseous VOCs and water vapor were introduced by passing air through a temperature-controlled bubble tower and they were then mixed with dilution air in a mixing chamber to reach the desired concentration. Relative humidity (RH) of the reaction gas was controlled at 50% at room temperature (298 K). The concentrations of benzene, toluene and *p*-xylene were all 50 ppm, while that of acetone was 250 ppm. The total flow rate was 2.0 L·min^−1^. All the reagents used in this study were analytically pure, obtained from the Beijing Chemical Corporation (Beijing, China).

The outlet concentrations of the VOCs, CO_x_ (CO and CO_2_), O_3_, NO_x_ (NO and NO_2_) were respectively detected. The VOCs in the gas stream were analyzed by an online gas chromatograph (Agilent, model 6890N, Santa Clara, CA, USA), equipped with a flame ionization detector (FID) and a 30.0 m × 320 μm HP-5 capillary column. The column temperature was 373 K and that of the detector was 423 K. The conversion of each VOC by non-thermal plasma (NTP) decomposition was defined by η, calculated according to Equation (1). The energy density (ED, J·L^−1^) was used to evaluate the validity of NTP technology, which was calculated according to Equation (2). In the present work, all the decomposition results were compared and discussed based on the ED. The outlet concentrations of CO and CO_2_ were analyzed by a gas chromatograph (Techcomp, model GC7890II, Beijing, China) equipped with an FID detector, a TDX-01 packed column, and a methane conversion oven prior to the detector. The CO_x_ selectivity was adopted to characterize the mineralization degrees of the VOCs in the present work, defined by SCO_x_ according to Equation (3). O_3_ was monitored by the Ozone Monitor (2B Technologies, 106-L, Boulder, CO, USA) according to the ultraviolet absorption method, while NO_x_ was monitored according to the *N*-(1-naphthyl) ethylene diamine dihydrochloride spectrophotometric method.

(1) $$\eta =\frac{C_{inlet}-C_{outlet}}{C_{inlet}}\times 100\text{\%},$$

(2) $$ED=\frac{U\times I}{Q}\times 60$$

(3) $${\;S}_{{\mathrm{CO}}_{x}}=\frac{\left(2-x\right)C_{\mathrm{CO}}+\left(x-1\right)C_{{\mathrm{CO}}_{2}}}{\sum n_{\mathrm{inlet}}C_{\mathrm{inlet}}^{\prime }\eta }\times 100\text{\%}$$

where *C_inlet_* and *C_outlet_* are inlet and outlet concentrations of the pollutant (ppm), respectively; *U* and *I* are the applied voltage (kV) and discharge current (mA), respectively, both of which can be automatically detected by the power supply equipment, checked before experiments; and *Q* is the flow rate of the reaction gas (L·min^−1^). C′_inlet_ is the inlet concentration of the pollutant (mg·m^−3^); CCO_2_ and CCO are the outlet concentrations of CO_2_ and CO (mg·m^−3^); and ninlet indicates the number of carbon atoms of the VOC.

### 2.3. Characterization

In order to clarify the mechanism of VOC degradation by NTP, the variations in the intermediate products were detected by a mass spectrometer (MS OmnistarTM, model GSD 301 O2, Preddvor, Slovenia) and Fourier transform infrared spectroscopy (FTIR, Nicolet 6700, Santa Monica, CA, USA). The mass spectrometer was used to explore the change of intermediate species during the reaction process. An electron multiplier and a tungsten filament were used to detect the specific charge, while the SEM voltage was controlled at 1.4 kV. The first mass was 44.5 u and the last mass was 100 u, except for *p*-xylene (110 u). The scan speed was 5 s. FTIR was used to investigate the change of the gas molecule functional group during the reaction process; all spectra were collected in the 3700–600 cm^−1^ frequency range at a resolution of 4 cm^−1^. In total, 16 scans were averaged for each spectrum, while the gathering time was 20 min.

The organic side products were collected using 5 mL methyl alcohol for 15 min, and then identified using gas chromatography–mass spectrometry (GC–MS, Shimadzu GC-2020, Kyoto, Japan) in electron impact mode (70 eV), equipped with a 60.0 m × 0.25 mm × 0.25 μm column (Agilent, HP5MS, Santa Clara, CA, USA), A 200 °C ion source was chosen. Argon was chose as the carrier gas and the flow rate was 1 mL·min^−1^.

The theory calculation was based on the Gaussian 09 package program combined with density functional theory (DFT). The molecular geometry of the VOCs was optimized using the DFT (B3LYP) method with a 6-311G++(d,p) basis set. The atomic partial charge, bond length, and electrostatic potential of HOMO orbits of the VOCs were calculated, and the corresponding results are listed in Table S1.

## 3. Results and Discussion

### 3.1. Effect of BTEX on the Acetone Removal Efficiency

**Figure 2:**
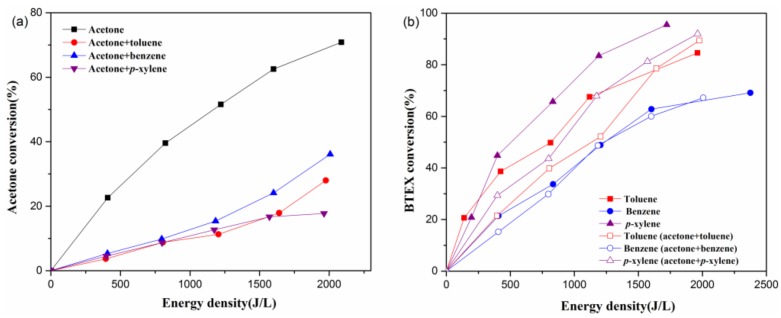
Effects of energy density on acetone (**a**) and BTEX conversion (**b**). Reaction conditions: acetone: 250 ppm, BTEX: 50 ppm, relative humidity (RH): 50%, and total flow: 2 L/min.

Acetone and BTEX conversions are studied as a function of energy density under the conditions of individual contaminants and two VOC mixtures. As shown in Figure 2a, when acetone is treated individually, its conversion increases from 0% to 71% with increasing ED. This result is similar to that of a previous study by Zheng et al. [22]. However, when acetone is degraded together with three kinds of BTEX under plasma treatment, its degradation efficiency reduces to less than 20% with an energy density of 1200 J·L^−1^, implying that BTEX has a significant negative impact on acetone conversion. The degree of influence of three different kinds of BTEX on acetone removal efficiency can be ordered as follows: toluene ≈ *p*-xylene > benzene.

However, it can be seen from Figure 2b that benzene conversion is hardly affected when it coexists with acetone, while the removal efficiencies of toluene and *p*-xylene decrease with the introduction of acetone. In addition, it is found that whether treated together with acetone or not, *p*-xylene conversion is much higher than that of toluene under the same ED. It is known that high-energy electrons ranging from 1011 to 1014 cm^−3^ contribute to VOC decomposition during the NTP process by two means: (1) directly breaking VOC molecules into organic fragments via high-speed collision; and (2) interaction with carrier gas molecules to generate large quantities of chemical reactive species (e.g., •O, •OH, O_3_, and metastable N_2_) for VOC removal [23]. On one hand, the original concentration of BTEX (50 ppm) in this study is far lower than that of acetone (250 ppm), and thus the probability of effective electron collision with acetone molecules may not change significantly after BTEX introduction. In other words, the types and concentration levels of active radicals in the NTP reactor should play a dominant role in contributing to BTEX inhibiting acetone degradation. On the other hand, the structural difference between the three kinds of VOCs is shown in the methyl group and its quantity in the benzene. Considering the efficiency difference shown in Figure 2, besides the benzene ring effect, it can be deduced that the hydrogen abstraction reactions on the methyl group may occur during BTEX degradation, also influencing the acetone plasma–chemical reactions, removal efficiency, and the degradation product.

### 3.2. Effect of the BTEX on CO_x_ Selectivity

**Figure 3:**
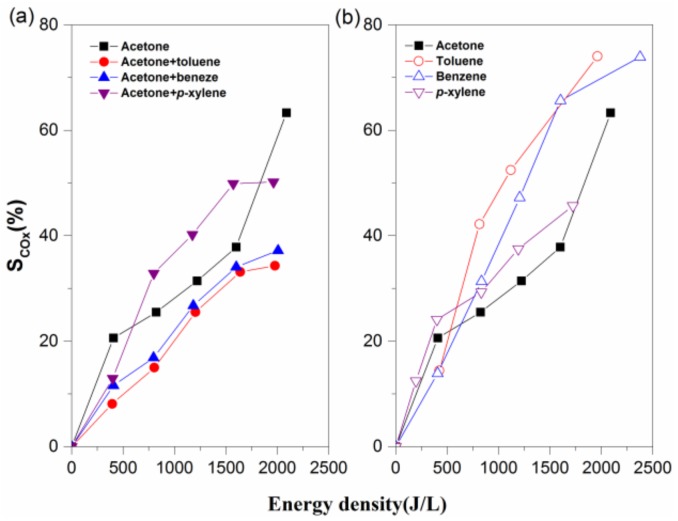
Effects of energy density on the Sco_x_ of the binary (**a**) and single-component (**b**) volatile organic compound (VOC) degradation processes. Reaction conditions: acetone: 250 ppm, BTEX: 50 ppm, RH: 50%, and total flow: 2 L/min.

Figure 3 shows the selectivity of CO_x_ as function of ED for the NTP process. It can be seen that in the case of BTEX and acetone together, the CO_x_ yield shows two stark divergences of trends: addition of *p*-xylene significantly promotes CO_x_ selectivity, thereby improving acetone mineralization; while CO_x_ production decreases after adding benzene or toluene into the NTP reaction system, meaning that more organic intermediates form. Figures S1 and S2 also provide the CO and CO_2_ selectivity under the binary and single-component VOC degradation processes. It is shown that more CO is generated when methylic BTEX and acetone are treated together, but the tendency is reversed for benzene introduction. By contrast, the selectivity of CO_2_ in the binary VOC degradation process is obviously lower than in single-component VOCs. Because more active free radicals are consumed when BTEX is treated together with acetone, the rate of CO_x_ mineralization and further oxidation of CO to CO_2_ is restricted. Equally important is that the hydrogen abstraction reaction on the methyl group occurred more easily with toluene or *p*-xylene degradation, which can lead to CO formation despite an adversity to the total oxidation of pollutants [24,25].

### 3.3. NO_2_ and O_3_ Formation Analysis

**Figure 4:**
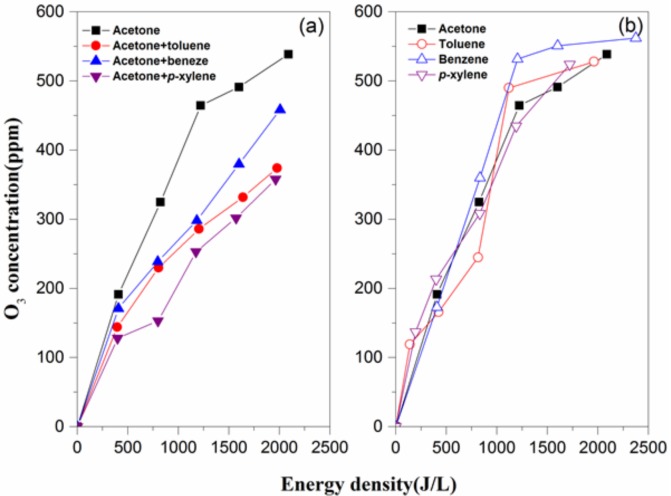
Effects of energy density on the O_3_ concentrations of the binary (**a**) and single-component (**b**) VOC degradation processes. Reaction conditions: acetone: 250 ppm, BTEX: 50 ppm, RH: 50%, and total flow: 2 L/min.

**Figure 5:**
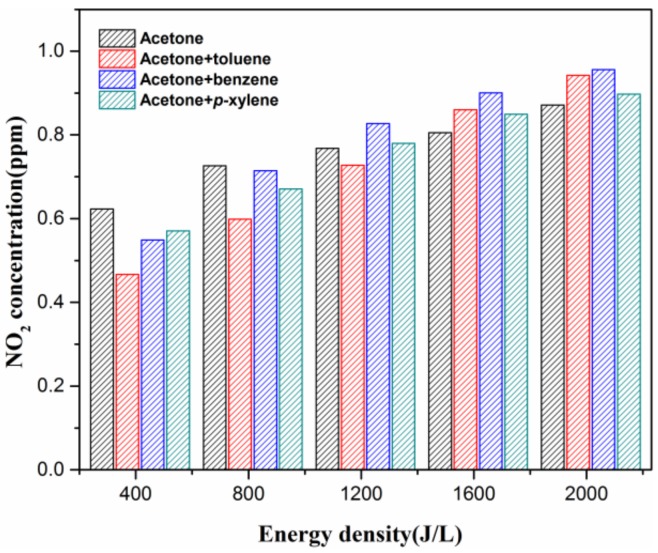
Effects of energy density on NO_2_ production. Reaction conditions: acetone: 250 ppm, BTEX: 50 ppm, RH: 50%, and total flow 2 L/min.

**Figure 6:**
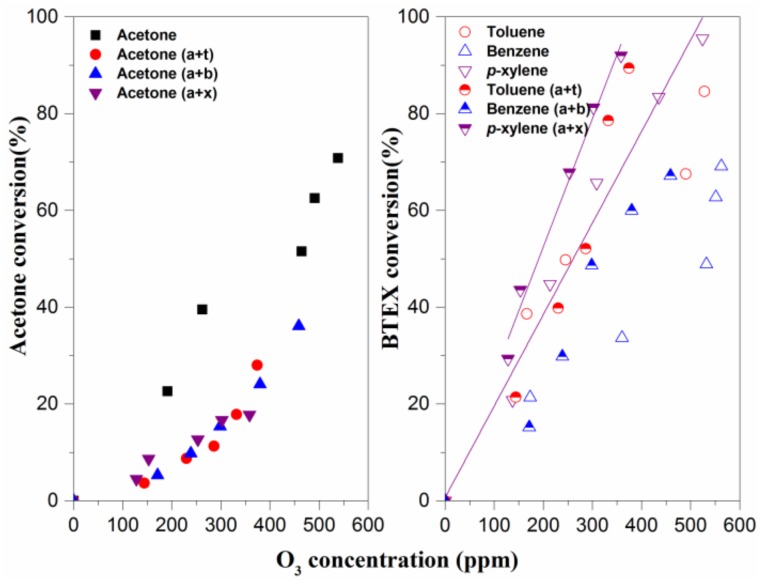
Effects of the O_3_ concentrations on acetone (**left**) and BTEX (**right**) conversions. Reaction conditions: acetone: 250 ppm, BTEX: 50 ppm, RH: 50% and total flow 2 L/min.

Figure 4 and Figure 5 provide the relationships between NO_2_, O_3_ concentrations and ED. As shown in the two figures, NO_2_ and O_3_ production monotonically increases with the increase of ED under both single and binary VOC mixture sets. Notably, the maximum NO_2_ concentration is less than 1 ppm, and NO is hardly detected in this study, so the effect of BTEX adding on NO_x_ formation may be negligible. It believed that O_3_ concentration is greater in the absence of VOCs at a relatively high ED (>200 J·L^−1^) considering ozone consumption by the oxidation of VOCs [26,27]. Therefore, even though VOC mixture degradation under the NTP process follows complex mechanisms, there may be some correlations between the ozone concentrations and pollutant treatment efficiency. Figure 6 gives the effect of O_3_ concentration on VOC concentrations. It can be seen that each BTEX conversion enhances significantly with increasing ED when degraded simultaneously with acetone, while acetone follows an inverse pattern. This phenomenon can be explained by the fact that BTEX oxidizes faster than acetone, since the former has a higher reaction rate constant with O_3_ and •OH [28]. Furthermore, there is significant positive linear correlation between O_3_ concentration and *p*-xylene conversion, while it is poor for other VOCs. Combined with the lowest yield of O_3_ for xylene-containing atmosphere degradation (Figure 5), it is reasonable to infer that O_3_ or its precursor from the plasma–chemical reactions can interact with the methyl group from the *p*-xylene molecule.

### 3.4. Transient Study during NTP Treatment

**Figure 7:**
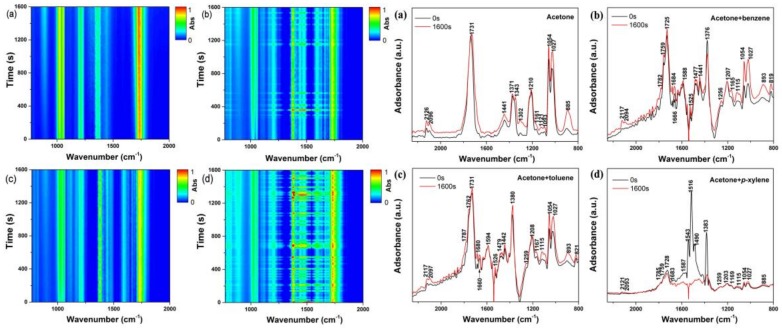
In situ FTIR results of the VOC degradation process at energy density (ED) = 1600 J/L: acetone (**a**); acetone–benzene (**b**); acetone–toluene (**c**); acetone–*p*-xylene (**d**).

Figure 7 and Table S1 show the detailed in situ FT-IR spectra of the acetone and acetone–BTEX mixture effluents under plasma treatment at an ED of 1600 J·L^−1^. When acetone is degraded alone, as the reaction time extends the bands at 1027 and 1054 cm^−1^, ascribed to the O-O stretching vibrations of the ozone, gradually increase. Besides, bands appear at 2126, 2096, 1731, 1441, 1373, 1343, 1302, 1210, 1161, 1121, 1093, and 885 cm^−1^ during the NTP process. Among them, the acetone species are characterized by the ν (C=O) at 1731 cm^−1^, δ (CH_3_) at 1441–1371 cm^−1^ and δ (C-C) at 1161 cm^−1^ [29,30]. As the reaction time continues, the intensity of the bands attributed to olefins (885 cm^−1^), formic acid (1093 and 1121 cm^−1^), oxalic acid (1302 cm^−1^), alcohol (1343 cm^−1^), and CO (2126 and 2096 cm^−1^) continuously increases on the spectra [16,31,32]. Narengerile’s research results indicate that HCOOH, HCHO, and soot are the main organic byproducts when acetone is decomposed by direct current (DC) plasma [18]. Therefore, the bands belong to the ν (C=O) stretching vibration of the aldehydes, which is usually located at around 1700 cm^−1^, and may be covered by the characteristic peak of acetone at 1730 cm^−1^.

For the two composite VOC atmospheres, the FTIR spectra in the range of 1800–1000 cm^−1^ are significantly different from those of the acetone system. The bands at 821 cm^−1^, 1475 cm^−1^, 1516–1526 cm^−1^, and 1660–1684 cm^−1^ represent the characteristic vibrational peaks of the benzene ring. The byproducts related to BTEX degradation consist of the phenolic hydroxyl (OH, 1252–1258 cm^−1^), aldehyde (CHO, 1759 cm^−1^), and carboxyl (COOH, 1787 cm^−1^) group, indicating that phenol, benzaldehyde, and benzoic acid may form under NTP treatment [33]. In the case of the acetone–xylene composite, the signal-to-noise ratio is significantly lower than for the acetone–benzene and acetone–toluene systems, meaning *p*-xylene becomes extremely unstable under the discharge condition. Additionally, the characteristic peaks of *p*-xylene at 1516 cm^−1^ disappear rapidly after treatment with NTP, suggesting its reaction rate is faster than for benzene and toluene. As shown in the Figure 7, production of O_3_ is greater as the reaction progresses, while the reverse result is found when benzene or toluene is treated alone (Figure S3). In general, ozone generated by NTP can be partly consumed by the oxidation of VOCs, but this is suppressed when acetone and BTEX coexist [27]. One possible explanation is that a harder degradation of acetone inhibits the reaction between BTEX and ozone, thereby augmenting O_3_ concentrations.

**Figure 8:**
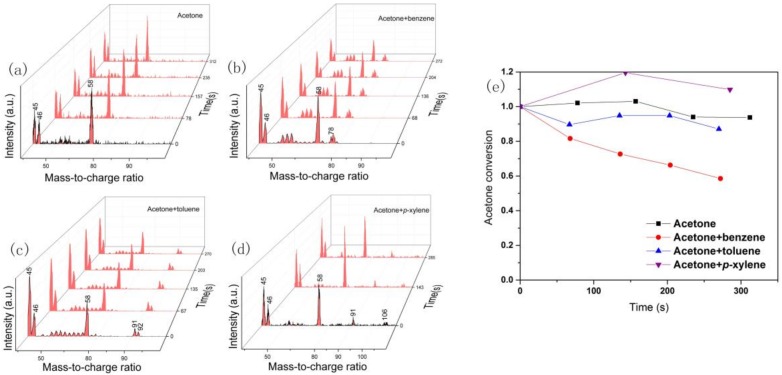
In situ mass spectrometry result of the outlet gas at ED = 1600 J/L: Acetone (**a**), acetone and benzene (**b**), acetone toluene (**c**) and acetone and *p*-xylene degradation (**d**) and acetone conversion (**e**).

Figure 8 shows the in situ MS spectrum of VOCs degradation at various discharge times. It is obvious that the production of *m*/*z* 45, 46, 58, 78, 91, and 106 occurs during NTP treatment. Among these, *m*/*z* 58, 78, 91, and 106 can be mostly attributed to acetone, benzene, toluene, and *p*-xylene, while *m*/*z* 45 and 46 can be attributed to the oxalic acid or fragment of carboxyl and formic acid, respectively. From this finding, together with the FTIR results, it can be speculated that a hydrogen abstraction from the benzene ring leads to a phenyl radical formation, which would react with •OH to form benzaldehyde and phenol or be further oxidized to benzoic acid. According to previous research, the hydrogen abstraction reaction from the methyl group will inevitably occur when toluene and acetone are decomposed by NTP and sequentially a reaction occurs with the excited •OH to form HCOOH, HCHO, and Cox [16,25]. It is reasonable to infer the proportion of formic acid in the carboxylic acid byproduct by the peak area ratio of *m*/*z* 46 to 45. When BTEX and acetone are treated together, the proportion of formic acid is clearly reduced compared with acetone alone, indicating that the acetone decomposition pathway is influenced by BTEX. Moreover, Figure 8 also gives the acetone degradation rate under different atmospheric conditions. It is clear that the benzene and toluene slightly accelerate the acetone removal but *p*-xylene lessens it. As is well known, benzene can be directly oxidized to various ring-opening byproducts under NTP, while for toluene or xylene, substituent groups on the benzene are more susceptible to decomposition, and benzene series byproduct generation occurs easily [15,34]. Therefore, more methyl group fragments derived from *p*-xylene or toluene decomposition may inhibit the acetone removal, which can also be decomposed to the methyl group and other products under NTP.

### 3.5. Organic Product Analysis by GC-MS

**Figure 9:**
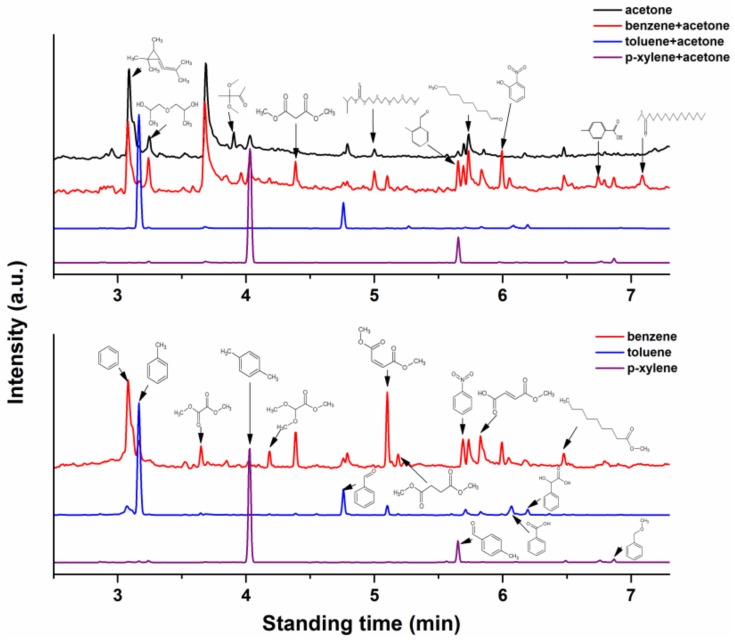
GC-MS results of the outlet gas at ED = 1600 J/L.

The organic byproducts of the effluent gas at ED of 1600 J·L^−1^ for single and binary VOCs systems are collected by GC-MS, as shown in Figure 9. The main byproducts during acetone degradation are annular and long-chain oxy-organics containing aldehyde, ethers, esters, etc. The number of organic byproducts, including both aromatic and ring-opening byproducts generated for benzene removal, is significantly greater than for toluene and *p*-xylene. Furthermore, it is seen that ring-opening byproducts show a significant decrease with the amount of methyl groups on the benzene ring. The major byproducts in the effluent gas of *p-*xylene treatment are benzene, toluene, 4-methyl benzaldehyde, 4-methylbenzoic acid, and benzyl methyl ether. When acetone and BTEX are treated together, the number of light ring-opening byproducts like dimethyl oxalate, methyl dimethoxyacetate, and dimethyl maleate is reduced, while the number of byproducts with more substituent groups on the benzene ring such as o-tolualdehyde, o-nitrophenol, and *p*-toluic acid rises. This phenomenon implies that instead of BTEX ring-opening, methylation and electrophilic substitution reactions in the benzene ring are favored under *co*-treatment of acetone and BTEX by NTP. Therefore, it can be concluded that the methyl group on acetone, toluene, and *p*-xylene decomposing into methyl fragments or radicals may have an immediate impact on the byproduct formation through a series of plasma chemical reactions.

### 3.6. Theoretical Analysis of Intermediates

In order to explore the mechanisms of the influence of BTEX on acetone decomposition, acetone and BTEX molecules underwent geometry optimization using the Gaussian 09 package program. Since VOCs are attacked by highly active oxygen species like •OH and •O through the electrophilic reactions, the attack position of active radicals on pollutants should have the greatest electron density in the HOMO orbital of the molecule, in accordance with the “frontier orbital” theory [35,36]. Therefore, the bond length, atomic charge, and electrostatic surface potential of total density and HOMO orbitals of the VOCs molecules are also calculated by the density functional theory, as shown in Figure S4 and Table S1. As seen in Figure S3, the positive electrostatic potential of acetone molecule is mainly concentrated in the region around the C1 and C3 atoms, whose atomic charge is −0.763 e on has the strongest electronegativity. Meanwhile, the C1–C2 bonds have the longest bond length (1.517 Å), so the cleavage of the methyl group would be the easiest step during the acetone decomposition process.

According to the ESP of HOMO orbitals for BTEX, it is found that introducing electron-donating methyl groups enhances the electron density of the benzene ring, facilitating attack by free radicals. Therefore, the substituent group position on the benzene ring would be easily occupied by electrophilic •OH radicals, resulting in further aromatic byproduct formation. On the other hand, as listed in Table S1, the carbon–carbon bond length on the benzene ring is shorter than that between the benzene ring and the substituent group. Thus, *p*-xylene with more methyl groups is more easily substituted through electrophilic reactions than other types of BTEX. Considering the higher electron density on the benzene ring, greater energy should be provided for ring-opening, thereby inhibiting the non-aromatic hydrocarbon byproducts, which may be a reasonable explanation for the GC-MS result. Consequently, after the –CH_3_ on toluene and *p*-xylene is removed from the aromatic ring, intermediates such as phenol, benzaldehyde, and benzoic acid would be generated. At the same time, the falling methyl fragment would have a negative impact on acetone decomposition because it may lead to broken acetone regeneration.

### 3.7. BTEX Effect on Acetone Degradation Pathway

The decomposition mechanism of acetone under various plasma treatment conditions has been reported and discussed by previous researchers [16,17,18,30]. The relative reaction rate constants involved are summarized in Table S2. When humidity is introduced into the reactor, the main reactive species are oxygen and hydroxyl radicals because O_3_ can act as a source of their formation, in accordance with Reactions (4)–(7) [37]:

(4) $$N_{2}\;+\;O_{2}\;+hv\to \;N_{2}+\;2O,$$

(5) $$H_{2}O\;+\;hv\to \;H+\;OH$$

(6) $$O_{3}\;+\;hv\to \;O_{2}+\;O$$

(7) $$H_{2}O\;+\;O\;\to \;2OH$$

High-energy electrons •OH and N_2_* directly dissociate acetone molecules into the fragment of •CH_3_ and CH_3_CO• via Reaction (8). Subsequently, the recombination of •CH_3_ and the oxygen species via several steps (Reactions (9)–(13)) can form CH_3_O_2_•, CH_3_O• and HCHO. The latter is further oxidized to HCO• by •OH or divided into CO and H (Reactions (14) and (15)) [38]. Additionally, HCO• is also the precursor of the CO and CO_2_, according to Reactions (16–19). Besides, a trace of NO_2_ can be detected, which may follow Reactions (20)–(23) [39].

(8) $$CH_{3}COCH_{3}\;\to \;CH_{3}+\;CH_{3}CO,$$

(9) $$CH_{3}CO\;+\;O\;\to \;CH_{3}+CO_{2}$$

(10) $$CH_{3}\;+\;O_{2}\;\to \;CH_{3}O_{2}$$

(11) $$CH_{3}\;+\;O_{3}\;\to \;CH_{3}O\;+\;O_{2}$$

(12) $$CH_{3}\;+\;O\;\to \;HCHO\;+\;H$$

(13) $$CH_{3}O+\;O_{2}\;\to \;HCHO\;+\;OH$$

(14) $$HCHO\;+\;OH\;\to \;HCO\;+\;H_{2}O$$

(15) HCHO → 2H+ CO

(16) HCO → CO+2H

(17) HCO + O → CO+ OH

(18) $$CO+\;O\;\to \;CO_{2}$$

(19) $$CO+\;OH\;\to \;CO_{2}+HO_{2}$$

(20) $$N_{2}\;\to 2N$$

(21) $$N\;+\;O_{3}\to \;NO+\;O$$

(22) $$CH_{3}O_{2}+\;NO\to CH_{3}O+\;NO_{2}+\;HO_{2}$$

(23) $$NO+\;O\to NO_{2}$$

**Figure 10:**
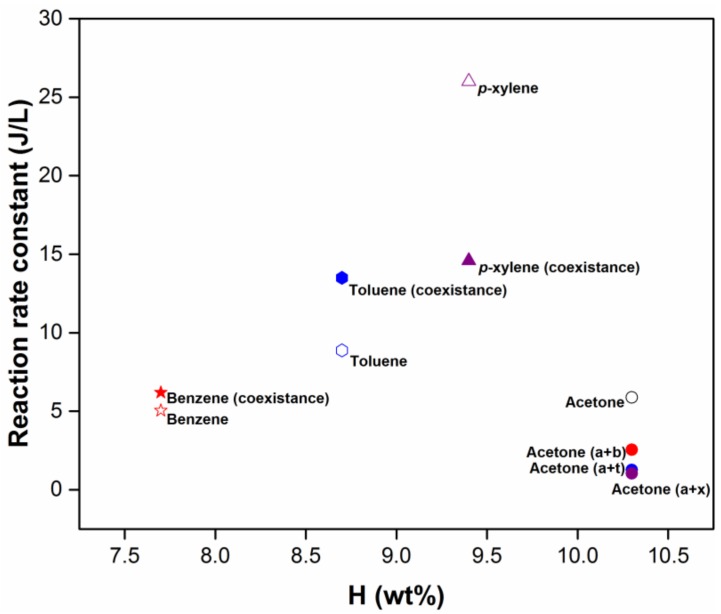
Reaction rate constants of the VOCs with O vs. the hydrogen weight fraction H (wt %).

On the basis of previous results, hydrogen abstraction, ring-opening, and isomerization are the major possible chemical pathways for BTEX oxidation by non-thermal plasma [11,15,40]. Nevertheless, each degradation pathway depends on the oxygen or hydroxyl radicals, and hence the acetone degradation is unavoidably influenced by BTEX because of radical consumption. On the one hand, in the light of a semi-empirical first-order kinetic model provided by previous studies, the reaction rate constant with O for the removal of *VOCs* in the NTP reactors can be predicted using Reactions (24) and (25) when the *VOC* removal is below 95% [26,41].

(24) $$O+\left[VOCs\right]\overset{k}{\to }\;products,$$

(25) $$\frac{\left[VOCs\right]}{{\left[VOCs\right]}_{0}}\;=e^{-ED/\beta }$$

where *k*, [*VOC*]_0_, and *β* are represent the reaction rate constant, original concentration of the VOC, and the regression parameter that is equal to −1/*k*, respectively. The *k* values calculated by different VOC atmospheres as a function of the hydrogen weight fraction are shown in Figure 10 and Table S3, because dehydrogenation reaction by O tends to be carried out on the methyl groups of VOCs undergoing treatment. The result shows that the order of BTEX reactivity is as follows: *p*-xylene > toluene > benzene, in agreement with the descending order of the methyl group amount. Furthermore, its impact trend on acetone reactivity implies that a greater hydrogen fraction in BTEX generally results in a greater loss of the acetone rate constant. On the other hand, since other possible pathways of VOC degradation may include ion–molecule, electrophilic and hydroxyl radical, and ozone reactions, ionization energy, proton affinity, and reaction constants (298 K) with the •OH and O_3_ of pollutants are referred to from the NIST and listed in Table 1 [42]. It is found that ozone reactivity is negligible, since reaction rate constants for ozone are far lower than for the hydroxyl radical. Both the sequence of •OH reaction constants and ease of ionization follow the (decreasing) order *p*-xylene > toluene > benzene > acetone, in consensus with the O reaction rate mentioned above. Thus, it is suggested that BTEX may be decomposed firstly rather than acetone. However, the order of proton affinities is as follows: acetone > *p*-xylene > toluene > benzene, implying acetone is chemically more reactive with electrophilic reagent than BTEX. It thus stands to reason that acetone can react with electrophilic intermediate species generated from hydrogen abstraction reaction on the methyl group under BTEX decomposition, thereby changing the original degradation pathway.

### 3.8. Roles of Methyl Species in the Acetone Degradation

**Figure 11:**
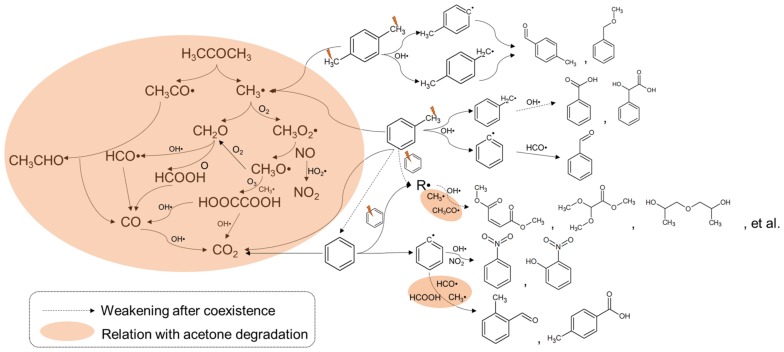
Schematic diagram of the influence mechanism of BTEX on the acetone degradation pathway.

In the plasma environment, acetone can be firstly decomposed into CH_3_• and CH_3_CO• by active radicals and high-energy electrons, as mentioned above (Equation (9)). Then, these two intermediates further go through a series of oxidation reactions to generate CO, CO_2_ and other byproducts by collisions with O or •OH radicals. As is well known, the methyl group (−CH_3_) usually plays a key role in the C1 chemistry, because it is the main precursor of HCHO, HCOOH, and other organic micro-molecules [43]. Therefore, it is believed that the fate of the hydrogen abstraction reaction on the methyl group largely determines the oxidation regime of acetone, as pointed by Alzueta’s research [24]. When acetone is treated together with methylic BTEX, amounts of intermediate products from methyl group reaction are inevitably generated and further participate in acetone degradation. For Reaction (9), there is an unfavorable shift in the equilibrium through the increase in the products, resulting in the decreased degradation efficiency of acetone, as well as that of toluene and *p*-xylene. This may be also a reasonable explanation for the immunity effect of benzene shown in Figure 2. Furthermore, considering a limited number of active radicals (mainly •OH), amounts of CH_3_• from acetone degradation would react with CO rather than being completely oxidized to CO_2_, leading to CO_2_ selectivity decreasing. Finally, the methyl group with electron-donating effect can promote the electron density of the benzene ring, which makes electrophilic •OH more easily consumed on the benzene ring than acetone, thereby intensifying aromatic byproduct formation. Therefore, a summary schematic diagram of the influencing mechanism of BTEX on the acetone degradation pathway is proposed, as shown in Figure 11.

## 4. Conclusions

In this research, the effect of BTEX on acetone degradation by non-thermal plasma was investigated in depth under negative DC corona discharge in humid air. The primary findings are as follows:

(1) BTEX has a significant negative impact on acetone conversion when they are treated together. The degree of influence of the three different kinds of BTEX on acetone removal efficiency can be ordered as follows: toluene ≈ *p*-xylene > benzene.
(2) *p*-xylene significantly promotes CO generation, thereby improving CO_x_ selectivity; while benzene or toluene decreases CO_x_ selectivity after coexistence with acetone.
(3) Based on the results of in situ experiments and GC-MS, it is found that the quantity of ring-opening byproducts is reduced, while the number of aromatic byproducts rises under abatement of mixture VOCs, indicating that instead of BTEX ring-opening, methylation, and electrophilic substitution reactions in the benzene ring are favored during decomposition.
(4) It is deduced that methyl radicals play an important inhibiting role in degradation efficiency and CO_x_ selectivity, which is mainly shown through: (1) unfavorable shifting of the equilibrium of acetone degradation reaction through the increase in the products; (2) reaction with limited active radicals to generate CO rather than highly oxygenated CO_2_; and (3) intensified side reactions on the benzene ring, thereby promoting aromatic byproduct formation.

## Figures and Tables

**Table 1:** The values for ionization energies, proton affinities, and reaction constants for reactions with the •OH and O_3_ of VOCs.

VOCs	IE (eV)	PA (kJ/mol)	*k*_OH_ (cm^3^ molecule^−1^s^−1^)	*k*_O3_ (cm^3^ molecule^−1^s^−1^)
Acetone	9.70	812.0	3.90 × 10^−14^	nf
Benzene	9.24	750.4	1.28 × 10^−12^	1.72 × 10^−22^
Toluene	8.83	784.0	6.16 × 10^−12^	3.9 × 10^−22^
*p*-xylene	8.44	794.4	1.52 × 10^−11^	nf

nf—not found in the NIST.

## Supplementary Materials

The following are available online, Figure S1: Effects of energy density on the Sco of binary (a) and single (b) component VOCs degradation process. Reaction conditions: Acetone: 250 ppm, BTEX: 50 ppm, 50% RH and total flow: 2 L/min, Figure S2: Effects of energy density on the Sco2 of binary (a) and single (b) component VOCs degradation process. Reaction conditions: Acetone: 250 ppm, BTEX: 50 ppm, 50% RH and total flow: 2L/min, Figure S3: In situ FTIR results of the single component VOCs degradation process (ED = 1600 J/L), Figure S4: The optimized acetone and BTEX structures, corresponding atomic charge and ESP of total density and HOMO orbital, Table S1: Main bond length of acetone and BTEX from theory calculations, Table S2: Relative reactions and rate constants, Table S3: Reaction rate constant (k) and β parameter.
