# Supplementary material for: Effects of BTEX on the Removal of Acetone in a Coaxial Non-Thermal Plasma Reactor: Role Analysis of the Methyl Group
Source: Molecules. 2018 Apr 5;23(4):890. doi: 10.3390/molecules23040890 (PMC6017784; doi:10.3390/molecules23040890)
Supplement: Supplementary file 1 [file molecules-23-00890-s001.pdf]

Supporting information

Effects of BTEX on the Removal of Acetone in A Coaxial Non-thermal Plasma (NTP) reactor：Role Analysis of the Methyl Group

Liyuan Hou, Xiang Li[*]*, Deyuan Xie, Tianle Zhu, Haining Wang*

School of Space and Environment, Beihang University, Beijing, 100191, PR China.

[*] Corresponding author: +86 10 82736373, E-mail address: xiangli@buaa.edu.cn (X. Li);

Table and Figure Captions

Table S1. Main bond length of acetone and BTEX from theory calculations.

Table S2. Relative reactions and rate constants.

Table S3. Reaction rate constant (*k*) and *β* parameter.

Figure S1. Effects of energy density on the Sco of binary (a) and single (b) component VOCs degradation process. Reaction conditions: Acetone:250 ppm, BTEX:50 ppm, 50%RH and total flow: 2L/min.

Figure S2. Effects of energy density on the Sco_2 of binary (a) and single (b) component VOCs degradation process. Reaction conditions: Acetone:250 ppm, BTEX:50 ppm, 50%RH and total flow: 2L/min.

Figure S3. In situ FTIR results of the single component VOCs degradation process (*ED*=1600 J/L).

Figure S4. The optimized acetone and BTEX structures, corresponding atomic charge and ESP of total density and HOMO orbital.

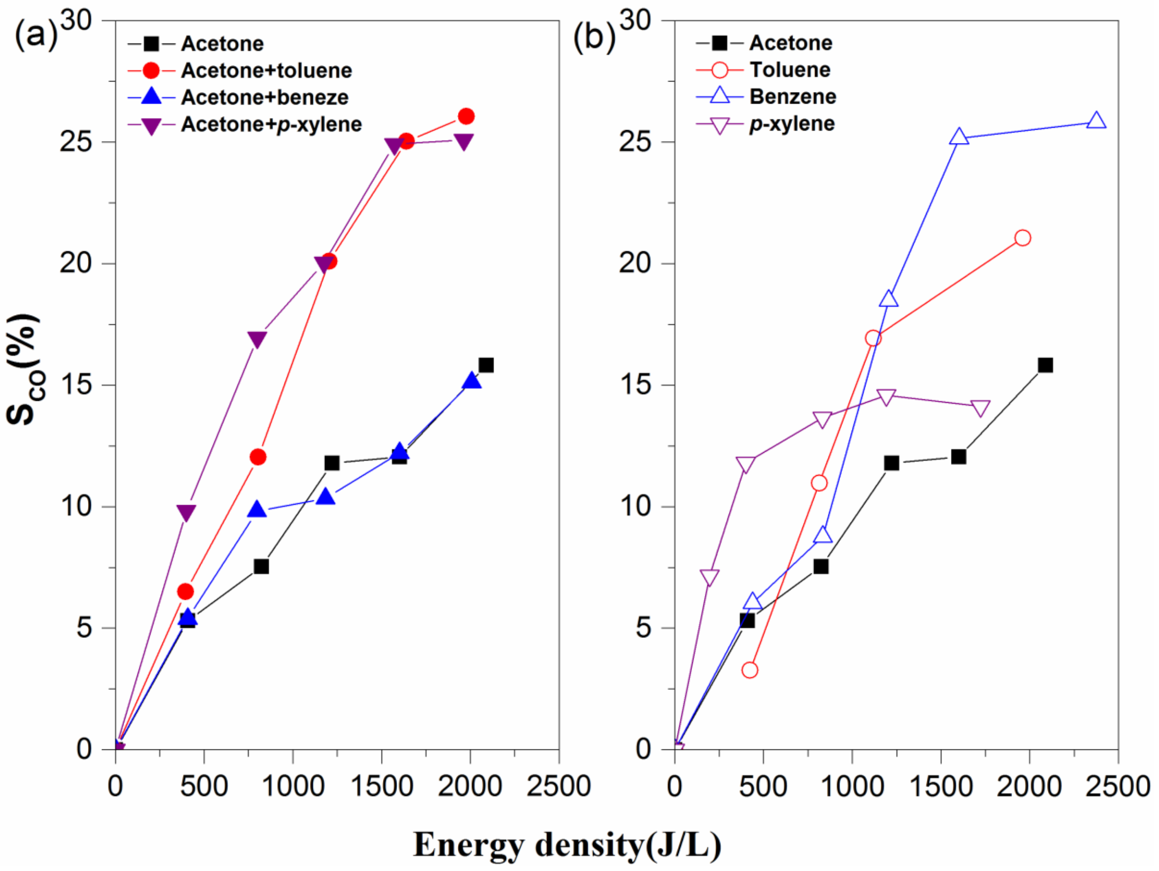

Fig. S1 Effects of energy density on the Sco of binary (a) and single (b) component VOCs degradation process.

Reaction conditions:Acetone:250 ppm, BTEX:50 ppm, 50%RH and total flow: 2L/min.

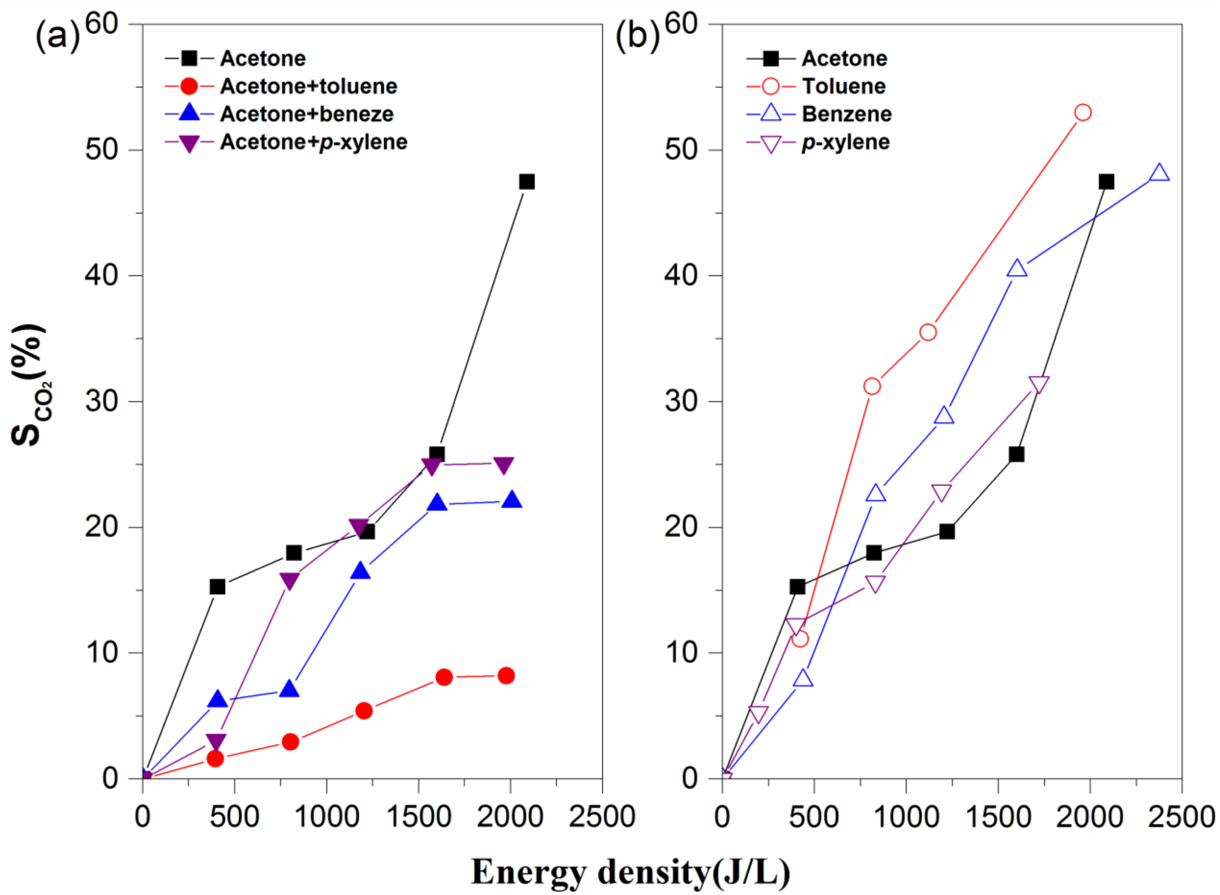

Fig. S2 Effects of energy density on the S_{CO_2} of binary (a) and single (b) component VOCs degradation process.

Reaction conditions:Acetone:250 ppm, BTEX:50 ppm, 50%RH and total flow: 2L/min.

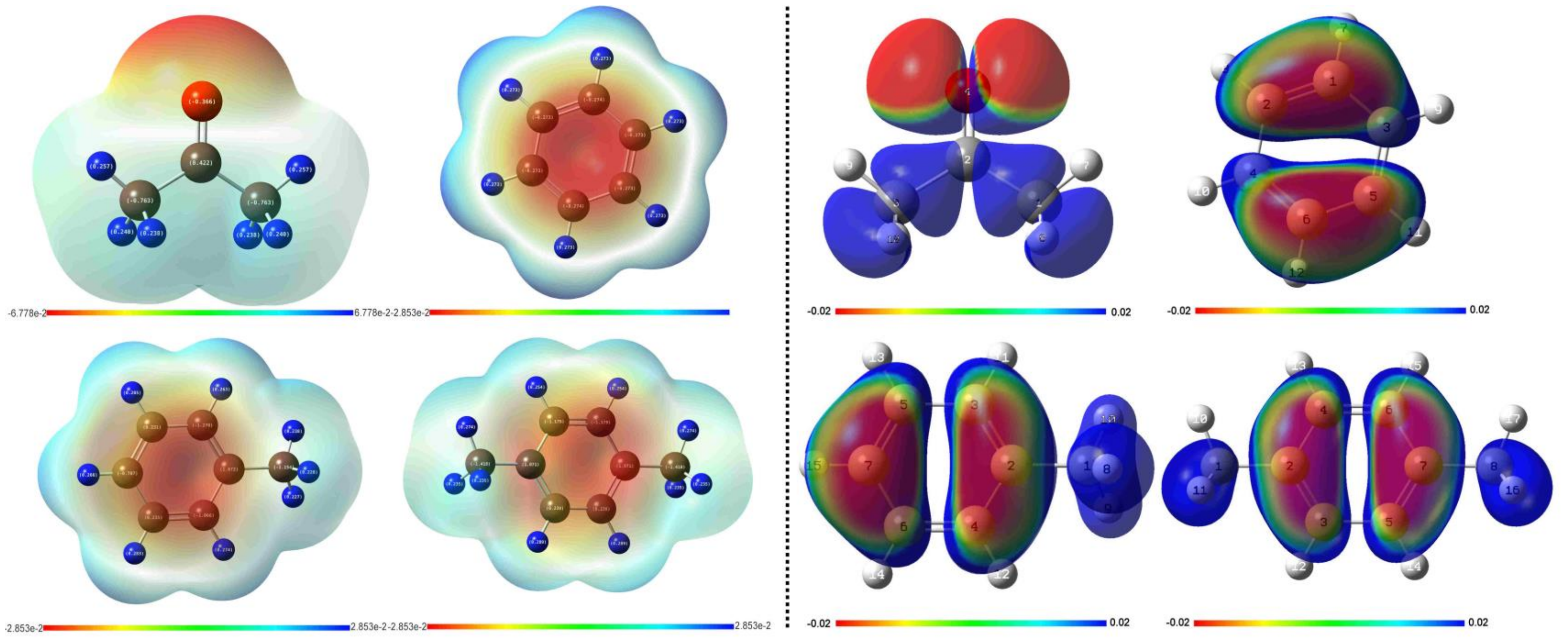

Fig. S3 The optimized acetone and BTEX structures, corresponding atomic charge and ESP of total density and HOMO orbital.

.

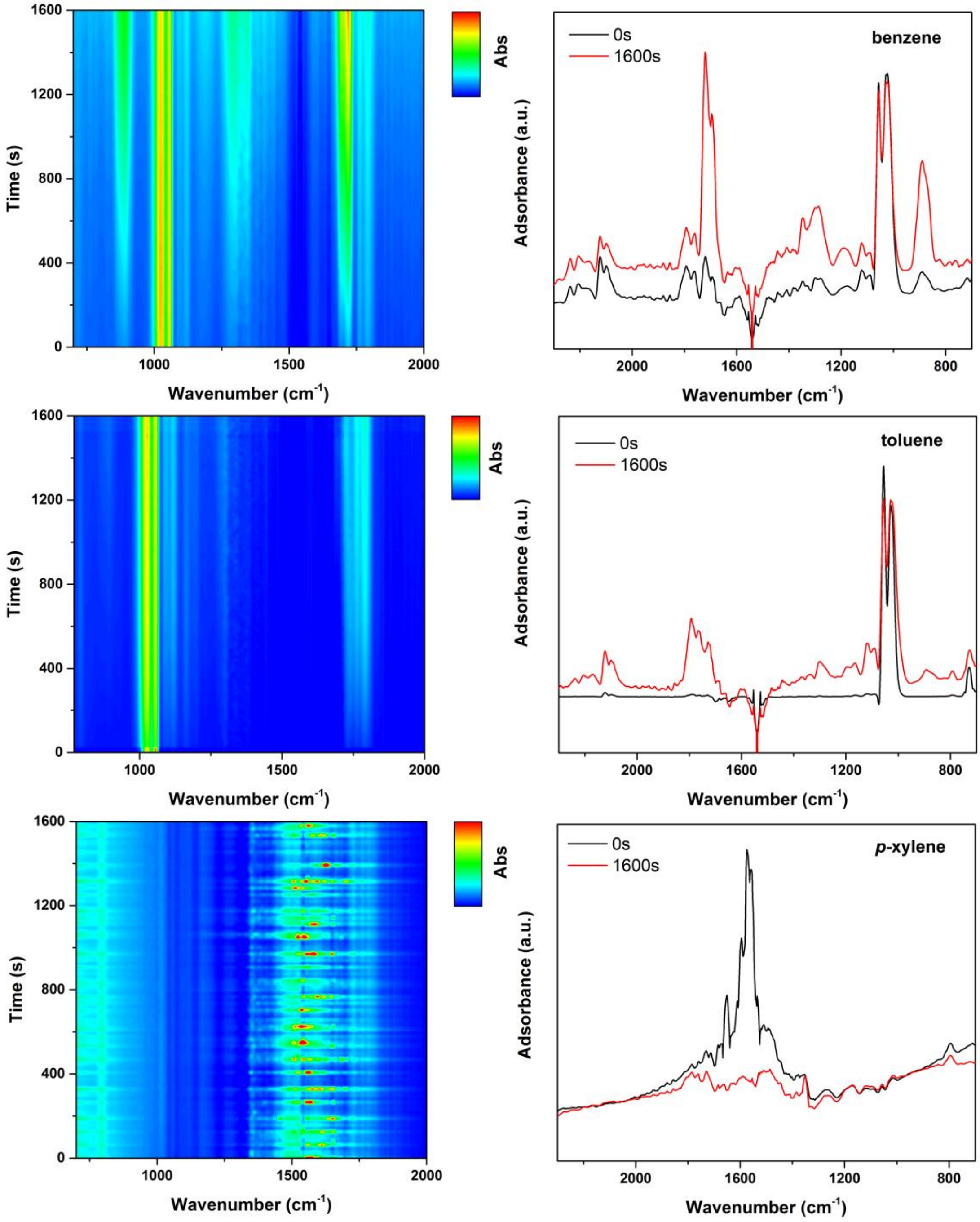

Fig. S4 In situ FTIR results of the single component VOCs degradation process (*ED*=1600 J/L).

Table S1. Main bond length of acetone and BTEX from theory calculations.

**Acetone**		**Benzene**		**Toluene**		***p*-xylene**	
Bond name	Bond length (Å)	Bond name	Bond length (Å)	Bond name	Bond length (Å)	Bond name	Bond length (Å)
C(2)-O(4)	1.212	C(1)-C(2)	1.394	C(1)-C(2)	1.510	C(1)-C(2)	1.513
C(1)-C(2)	1.517	C(1)-H(7)	1.084	C(1)-H(8)	1.093	C(1)-H(9)	1.094
C(1)-H(6)	1.094			C(1)-H(9)	1.093	C(1)-H(10)	1.091
C(1)-H(7)	1.089			C(1)-H(10)	1.096	C(1)-H(11)	1.094
C(1)-H(8)	1.095			C(2)-C(3)	1.400	C(2)-C(3)	1.406
				C(3)-C(5)	1.394	C(3)-C(5)	1.394
				C(3)-H(11)	1.086	C(3)-H(12)	1.084
				C(5)-C(7)	1.394	C(5)-C(7)	1.406
				C(5)-H(13)	1.085	C(5)-H(14)	1.084
				C(7)-H(15)	1.084		

Table S2. Relative reactions and rate constants

Reactions	Rate constant at 298K($cm^3 molecule^{-1} S^{-1}$)
CH_2O+OH▪→HCO▪+ H_2O	6.03×10^{-12}
CH_2O+O▪→HCOOH	1.01×10^{-11}
CH_3CO▪+ HCO▪→CH_3CHO+CO	9.04×10^{-12}
CH_3▪+O▪→CH_2O + H▪	5.66×10^{-13}
CH_3▪+O_2→CH_3O_2▪	1.08×10^{-12}
CH_3O_2▪+ NO→CH_3O▪+ NO_2+ HO_2▪	7.6×10^{-12}
CH_3O▪+ O_2→HCHO+HO_2	1.15×10^{-9}
HCHO+ OH▪→HCO+H_2O	6.03×10^{-12}
CH_3CHO+O_3→CH_3COOH	2.70×10^{-11}
HCO▪+ OH▪→CO+H_2O	1.02×10^{-14}
CO+ OH▪→CO_2+HO_2	1.55×10^{-13}*
Benzene+ OH▪→Products	1.28×10^{-12}*
Toluene+ OH▪→Products	6.16×10^{-12}*
1,4-Dimethylbenzene+ OH▪→Products	1.52×10^{-11}*

* Found in the NIST

Table S3. Reaction rate constant (*k*) and *β* parameter

VOCs	$k \times 10^4$ (J/L)	β (L/J)	R^2	H (wt%)
Acetone	5.88	1702	0.999	10.3
Acetone (a+b)	2.55	3914.2	0.942	10.3
Acetone (a+t)	1.26	7931.8	0.974	10.3
Acetone (a+x)	1.03	9723.1	0.973	10.3
Benzene	5.04	1982.3	0.949	7.7
Benzene (a+b)	6.19	1616.7	0.993	7.7
Toluene	8.87	1127.4	0.975	8.7
Toluene (a+t)	13.5	738.9	0.928	8.7
p-xylene	26	384.63	0.938	9.4
p-xylene (a+x)	14.6	687.04	0.962	9.4
